# HANDS: an RGB-D dataset of static hand-gestures for human-robot interaction

**DOI:** 10.1016/j.dib.2021.106791

**Published:** 2021-01-30

**Authors:** Cristina Nuzzi, Simone Pasinetti, Roberto Pagani, Gabriele Coffetti, Giovanna Sansoni

**Affiliations:** Department of Mechanical and Industrial Engineering (DIMI), University of Brescia, Brescia, Italy

**Keywords:** Hand-Gesture Recognition, Human-Robot Interaction, Classification, Object Detector

## Abstract

The HANDS dataset has been created for human-robot interaction research, and it is composed of spatially and temporally aligned RGB and Depth frames. It contains 12 static single-hand gestures performed with both the right-hand and the left-hand, and 3 static two-hands gestures for a total of 29 unique classes. Five actors (two females and three males) have been acquired performing the gestures, each of them adopting a different background and light conditions. For each actor, 150 RGB frames and their corresponding 150 Depth frames per gesture have been collected, for a total of 2400 RGB frames and 2400 Depth frames per actor.

Data has been collected using a Kinect v2 camera intrinsically calibrated to spatially align RGB data to Depth data. The temporal alignment has been performed offline using MATLAB, aligning frames with a maximum temporal distance of 66  ms.

This dataset has been used in [1] and it is freely available at http://dx.doi.org/10.17632/ndrczc35bt.1.

## Specifications Table

**Table:** 

Subject	Computer Science, Human-Computer Interaction
Specific subject area	Image Processing, Hand-Gesture Recognition
Type of data	Images Annotations
How data were acquired	Images were acquired using a Kinect v2 sensor connected to a machine equipped with Ubuntu 16.04 and using ROS packages libfreenect2 and iai_kinect2 to obtain the frames in the raw format of *rosbags*. The sensor has been intrinsically calibrated in advance to obtain a spatial alignment between the RGB frames and the Depth frames.
Data format	Raw
Parameters for data collection	The subjects were chosen considering gender equality, skin color, and the shape of the hands. The acquisitions have been conducted considering different light conditions of the scene (both artificial and natural), different backgrounds, different hand positions, and orientations.
Description of data collection	The *rosbags* containing the raw data were processed using MATLAB 2017b to convert the raw data as images (uint8 for the RGB ones and uint16 for the Depth ones). Using the timestamp of each frame, RGB and Depth frames have been temporally aligned with a maximum time difference between RGB and Depth frames of 66 ms.
Data source location	Brescia, Italy, University of Brescia, Department of Mechanical and Industrial Engineering, Latitude: 45.562959, Longitude: 10.23156
Data accessibility	Repository name: Mendeley Data Data identification number: 10.17632/ndrczc35bt.1 Direct URL to data: http://dx.doi.org/10.17632/ndrczc35bt.1
Related research article	C. Nuzzi, S. Pasinetti, R. Pagani, S. Ghidini, M. Beschi, G. Coffetti, and G. Sansoni, “MEGURU: a gesture-based robot program builder for Meta-Collaborative workstations,” *Robotics and Computer-Integrated Manufacturing,* vol. 68, p. 102085, 2021. doi: https://doi.org/10.1016/j.rcim.2020.102085

## Value of the Data

• This dataset comprehends 29 classes of static hand-gestures in the form of both RGB images and Depth images, spatially and temporally aligned. The annotations, in the form of bounding box coordinates, are provided in a separate file.
• The data is valuable for the field of Computer Vision, especially for the tasks of hand-gesture recognition, human-machine interaction, and hand-pose recognition.
• The data provided can be used to train Deep Learning models to recognize the gestures in the dataset using only a single modality (RGB or Depth) or both at the same time. It is also useful as a reference dataset for benchmarking models.
• The provided data is temporally and spatially aligned. We also provide our scripts to process it.

## 1 Data Description

Gesture recognition is an important field of study for human-machine interaction research since it is one of the most natural methods for humans to communicate. Thanks to Machine Learning and Deep Learning methods, nowadays the recognition of the hands even in cluttered environments has been made easy, provided that a suitable amount of data to train the model is used. Therefore, different datasets have been made public by the research community during the years, often with specific characteristics that made them more suitable for some models and applications with respect to others. For example, datasets suitable for CNN models are not suitable for Object Detector models and vice versa. In fact, most datasets contain color images where the hands are the only object present in them (with both simple and cluttered backgrounds), making them suitable for CNN models or segmentation techniques such as the ones in [2], [3]. Multi-modal datasets are also available, such as the one presented in [4], [5], [6], [7] where static gestures have been collected in the form of RGB and Depth frames spatially and temporally aligned. The drawback of these datasets is that only one gesture per image is shown and the hand is very close to the camera, making this dataset not suitable for Object Detector models that aim to detect gestures in big images also picturing cluttered environments.

Authors of [8] proposed an RGB-D dataset of both static and dynamic body gestures, where simple background and artificial light conditions have been adopted to facilitate the recognition process. However, the proposed body gestures are mostly dynamic and complex, more suited for games or computer interactions and not for industrial applications, where simple and easy gestures are needed.

The HANDS dataset proposed in this work differs from the others because it has been purposely created for Object Detector models; hence, the provided RGB and Depth images are big and a large portion of the cluttered background is present. HANDS is an improved version of the dataset adopted in [9], and it is composed of 15 static hand-gestures represented in Table 1, which are:

• Digits from 1 to 9 (from Table 1 (a) to Table 1 (i)), the 0 digit is represented by the gesture in Table 1 (j).
• A Span gesture (Table 1 (k)).
• A directional gesture pointing left or right accordingly (Table 1 (l)).
• Two-hands gestures that represent intuitive commands (from Table 1 (m) to Table 1 (o)).

**Table 1:** Front right-hand gestures variants as in [1].

Gestures from Table 1 (a) to Table 1 (k) have two variants: one performed with the right hand and one performed with the left hand. The gesture in Table 1 (l) has four variants, according to the direction the hand is pointing to and the hand used: right-hand pointing left (back facing the camera) and right (palm facing the camera), left-hand pointing left (palm facing the camera) and right (back facing the camera). Gestures performed with both hands have only one variant (from Table 1 (m) to Table 1 (o)). Thus, considering all the variants, the HANDS dataset comprehends 29 different classes.

To correctly assign the labels to each variant, each class is named after the gesture name (e. g. “Nine”) and has a suffix to identify the variant of the gesture, following this structure:

• V is used for vertical gestures, while H is used for horizontal gestures.
• F identifies the version of the gesture where the front of the hand is facing the camera, while B identifies the version where the back of the hand is facing the camera.
• R is used for right-hand gestures, while L is used for left-hand gestures.

Two female subjects and three males have been acquired performing the gestures. For each of them, a different background has been chosen to increase the variability of the dataset (Table 2). To speed up both the acquisition and the labeling process, the subjects were told to perform the same gesture with both hands. The performing style of the subjects was twofold: subjects 1, 4, and 5 performed the gestures standing in the same position front-facing the camera, the hands moving (i) towards the camera to acquire different depth values of the hands, and (ii) laterally at shoulder height, carefully rotating the hands to still be able to recognize the correct gesture. On the other hand, subjects 2 and 3 performed the gestures while moving randomly in the field of view of the camera, also moving the hands towards the camera and laterally. Light conditions in the scenes were also different: in the scenes used for subject 4 only artificial light was used, while for the others a combination of artificial and natural light has been adopted.

**Table 2:** Examples showing the different subjects involved in the study performing the gestures as in [1]. Color and Depth frames are shown for each subject.

After carefully selecting the most representative images for every gesture and actor, discarding the blurred and occluded ones, we selected a total of 150 RGB frames and their corresponding 150 Depth frames per gesture for each subject. The dataset is composed as follows:

• Subject 1 (M): 2400 RGB images and their corresponding 2400 Depth images, 150 + 150 frames per gesture. Cluttered background with artificial light only, subject standing still.
• Subject 2 (F): 2400 RGB images and their corresponding 2400 Depth images, 150 + 150 frames per gesture. Cluttered background with a combination of natural and artificial light, subject moving.
• Subject 3 (M): 2400 RGB images and their corresponding 2400 Depth images, 150 + 150 frames per gesture. Cluttered background with a combination of natural and artificial light, subject moving.
• Subject 4 (F): 2400 RGB images and their corresponding 2400 Depth images, 150 + 150 frames per gesture. Grey uniform background with artificial light only, subject standing still.
• Subject 5 (M): 2400 RGB images and their corresponding 2400 Depth images, 150 + 150 frames per gesture. Cluttered background with intense natural light from behind, subject standing still.

We provide the following data:

• For each subject a separate compressed file named “*SubjectN.zip*”, where N identifies the number of the subject with respect to Table 2. Each compressed file contains two directories: a Color directory containing only its color frames, and a Depth directory containing only the corresponding depth frames. Each frame has a resolution of *960* *×* *540 px* and the file names are composed of a number and a suffix identifying if it was a color or a depth frame (e. g. 028_color.png and 028_depth.png).
• MATLAB tables of each subject, containing the path of each color and depth frame and the bounding box positions in MATLAB format.
• The base MATLAB table used with a first empty row, called base_table.mat.
• Annotation files that reproduce the MATLAB tables in a textual format. These are used to build record files after correctly modifying the box coordinates using the script “*RecordCreate”*.

Each annotation file must be modified by users according to the path where they save the frames after extracting them from the compressed files. This can be done easily using the “*Find and Replace*” option of any common text editor.

To aid users to reproduce our elaboration in MATLAB we provide the following scripts:

• Bags2Imgs: a MATLAB script to convert the *rosbags* acquired using ROS, perform the temporal alignment, and save the raw data as images.
• Table2Txt: a MATLAB script to convert a MATLAB Labeling Session into our table format, perform a shuffle of the obtained dataset and write it into a textual file. This script is the one we used to obtain the provided MATLAB tables and the textual files.

To users who do not want to reproduce our labeling session but only need the data for other purposes such as the training of a network, we provide an example script in Python named RecordCreate that converts the annotation files into a TensorFlow record. This script is also useful to convert the bounding box coordinates from MATLAB to TensorFlow format.

**Fig. 1:**
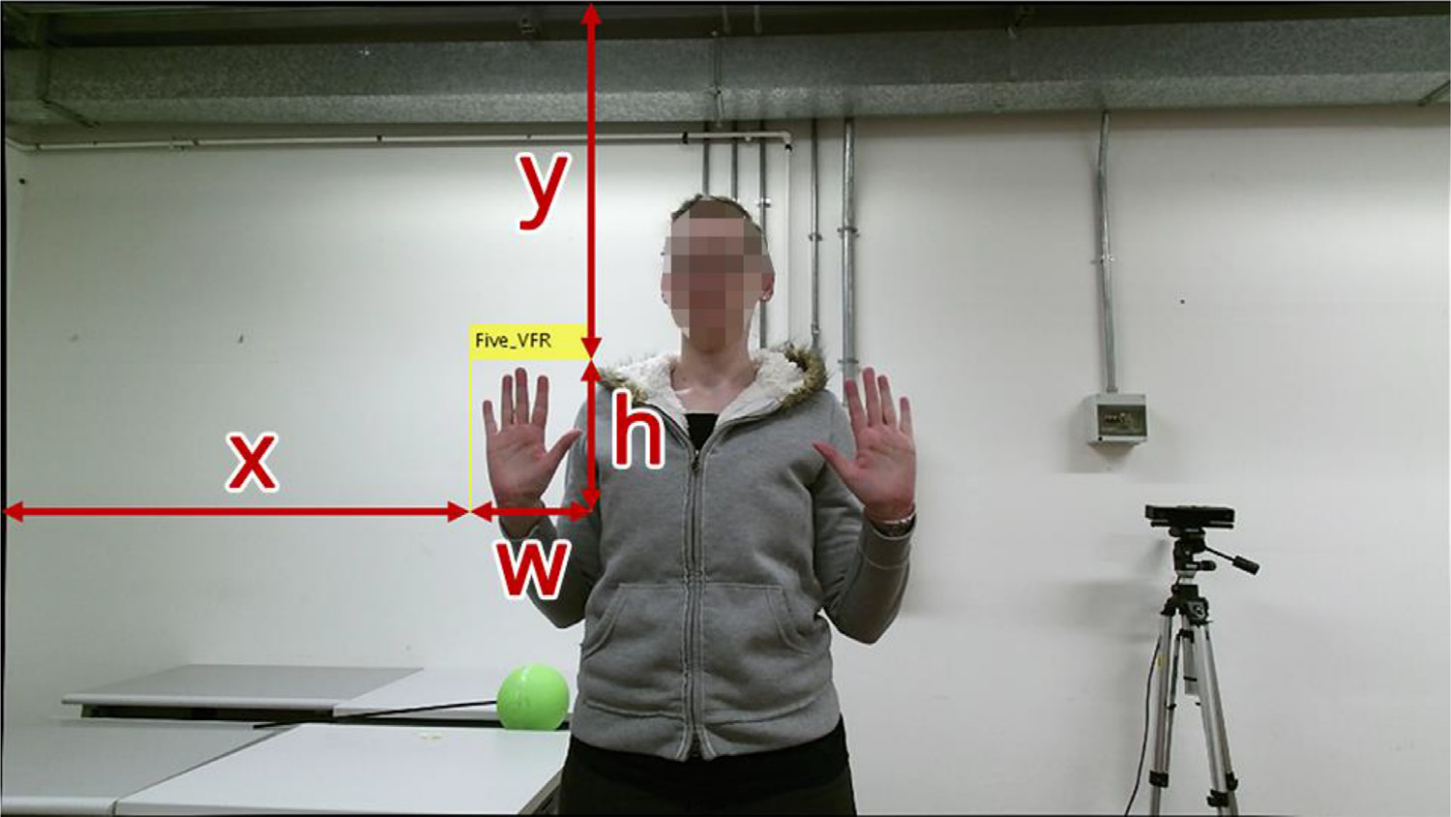
Example of how MATLAB creates the bounding box coordinates. The top-left point is the reference point for coordinates x and y.

It is worth noting that MATLAB coordinates of bounding boxes are defined as an array of four coordinates *(x, y, w, h)* defined as in Fig. 1, so it may be necessary to convert them according to the bounding boxes definition of choice. This is done by RecordCreate to convert them to TensorFlow format.

## 2 Experimental Design, Materials and Methods

The dataset has been acquired using a Kinect v2 sensor [10] intrinsically calibrated to spatially align the depth and RGB frames. Since the sensor acquires the frames at different times, a temporal alignment procedure of the frames has been carried out afterward. The images have been acquired frame by frame at 30 fps using ROS [11], libfreenect2 [12], and iai_kinect2 [13] ROS packages. We saved each acquisition into a *rosbag* and processed them afterward using MATLAB 2017b.

**Fig. 2:**
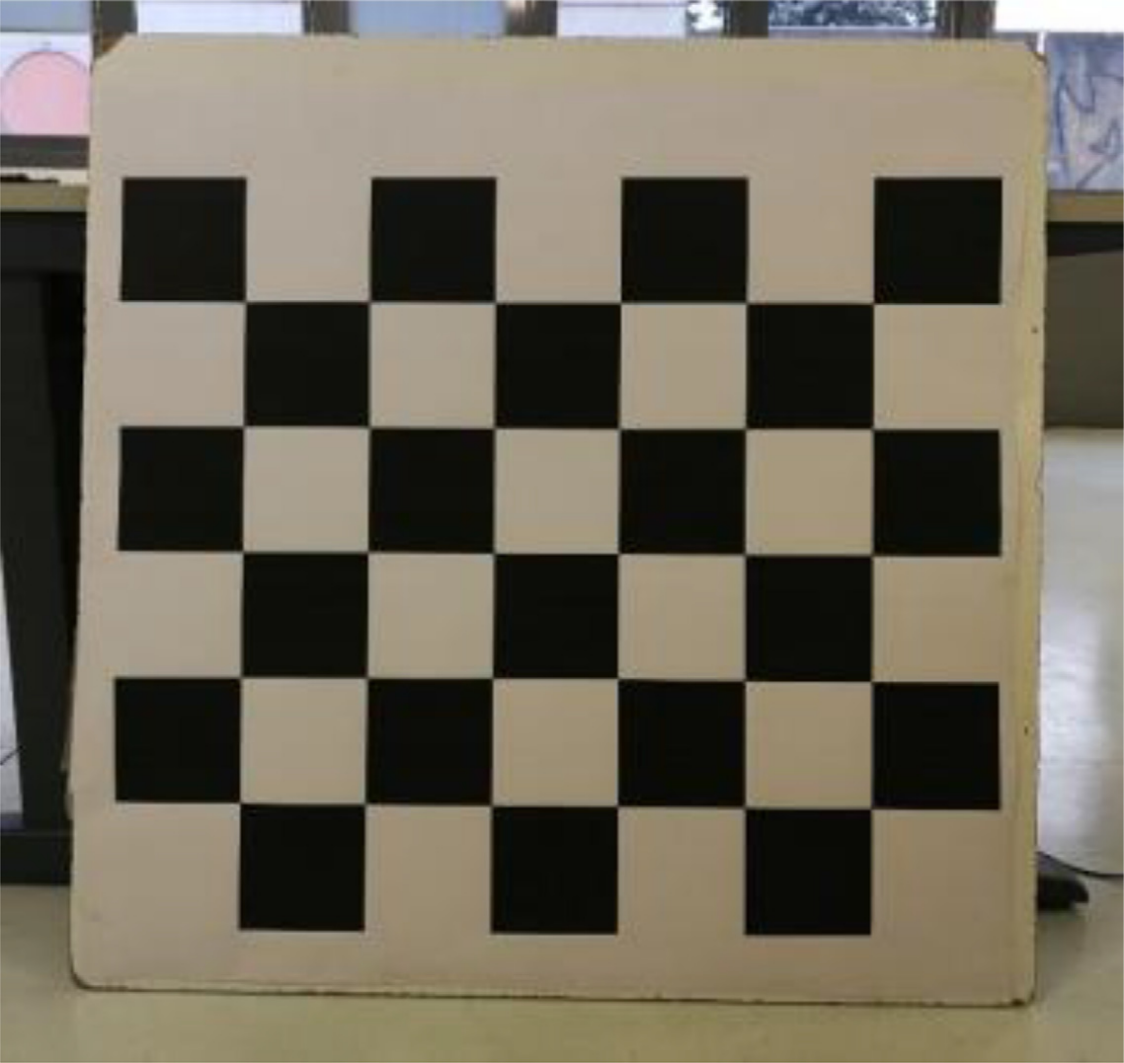
The chessboard used to calibrate the Kinect v2 sensor. It was printed and glued on a wooden support to move it around easily during the calibration process.

To achieve the spatial alignment, it is required that the sensor used is intrinsically calibrated to properly align the color information on top of the depth information. This procedure has been carried out in advance using the calibration tool of the iai_kinect2 package: a chessboard with a grid of *7* *×* *6* squares of *120* *mm* has been used to calibrate the sensor (Fig. 2).

It is also worth noting that the intrinsic calibration allows the iai_kinect2 package to correct the camera distortions while acquiring the frames: because of this, we acquired the corrected frames with a resolution of *960* *×* *540 px* (ROS topics kinect2/qhd/image_color_rect and kinect2/qhd/image_depth_rect respectively).

To achieve the temporal alignment, each acquisition has been elaborated using MATLAB by the “*Bags2Imgs*” script:

• To perform the calibration, we must identify a reference stream to which refer the others. We select the reference frame from the list of messages according to which one of the two streams contain fewer messages.
• Then, we find the corresponding frame in the other stream that has the minimum temporal distance from the selected reference frame.
• If the temporal distance is less than *66* *ms*, the couple is valid and properly saved.

## Ethics Statement

Informed consent has been obtained from each subject participating in the study, following the ethics guidelines. Furthermore, since the RGB frames contain identifiable traits of the subjects, to better ensure their privacy while still providing useful data for gesture recognition research we pixelated each face from the color frames using the automatic procedure described in [14]. We also manually checked each frame to make sure that the corrections do not affect the hands and that each identifiable feature has been anonymized. The images that the automatic procedure failed to pixelate were manually anonymized using Photoshop pixelate function. It is worth noting that since the depth frames do not contain sensitive information about the subject's identity, these data have not been modified.

## CRediT Author Statement

**Cristina Nuzzi:** Conceptualization, Methodology, Software, Data curation, Writing-Original draft preparation; **Simone Pasinetti:** Validation, Methodology, Software; **Roberto Pagani:** Investigation, Resources; **Gabriele Coffetti:** Investigation, Resources; **Giovanna Sansoni:** Supervision, Writing-Reviewing, and Editing.

## Declaration of Competing Interest

The authors declare that they have no known competing financial interests or personal relationships which have, or could be perceived to have, influenced the work reported in this article.

## Data Availability

HANDS: a dataset of static Hand-Gestures for Human-Robot Interaction (Original data) (Mendeley Data)
